# A Centenarian

**Published:** 1883-02

**Authors:** 


### A Centenarian.

From *The Lancet* we learn that Miss Sarah Apted, residing at Fern Villa, Garlands-road, Redhill, attained on the 20th of November the great age of 100 years, she having been born at Reigate, on December 2, 1782. There is no doubt about the authenticity of her age, as the entry of her birth and the date may be found in the books of Reigate parish church. She enjoys very fair health, gets up and dresses every day, has a good appetite, and occasionally takes and enjoys a glass of port. She has only one useful eye, having lost the other, as she says, "from inflammation, in the month," meaning in the first month of her life.

